# Supplementary material for: The possibility of sports industry business model innovation based on blockchain technology: Evaluation of the innovation efficiency of listed sports companies
Source: PLoS One. 2022 Jan 25;17(1):e0262035. doi: 10.1371/journal.pone.0262035 (PMC8789155; doi:10.1371/journal.pone.0262035)
Supplement: S2 Table — This table presents the means, standard deviations, and correlations. Most significant correlations (e.g., between investment income and proportion of R&D personnel in total employees, and between patent value and R&D expenditure as a proportion of main business income) are as expected. (PDF) [file pone.0262035.s002.pdf]

**Table 2: Summary Statistics and Correlations**

| Variables | Mean | S.D. | 1 | 2 | 3 |
|---|---|---|---|---|---|
| 1. Investment income | 22.5371 | 4.606 | | | |
| 2. Patent value | 4.77 | 0.525 | 0.18 | | |
| 3. Proportion of R&D personnel in total employees | 5.3481 | 0.6 | 0.54** | 0.64* | |
| 4. R&D expenditure as a proportion of main business income | 19.0484 | 6.339 | 0.46* | 0.72* | 0.346* |

***p<0.01; **p<0.05; *p<0.1
