# Supplementary material for: The possibility of sports industry business model innovation based on blockchain technology: Evaluation of the innovation efficiency of listed sports companies
Source: PLoS One. 2022 Jan 25;17(1):e0262035. doi: 10.1371/journal.pone.0262035 (PMC8789155; doi:10.1371/journal.pone.0262035)
Supplement: S1 File — (PDF) [file pone.0262035.s003.pdf]

AJE®

# Editing Certificate

This document certifies that the manuscript

**The possibility of sports industry business model innovation based on blockchain technology: Evaluation of the innovation efficiency of listed sports companies**

prepared by the authors

**Chenchen Lv, Yifeng Wang, Chai Jin**

was edited for proper English language, grammar, punctuation, spelling, and overall style by one or more of the highly qualified native English speaking editors at AJE.

This certificate was issued on **July 28, 2021** and may be verified on the AJE website using the verification code **E047-A3F4-203A-DC22-1AED**.

Neither the research content nor the authors' intentions were altered in any way during the editing process. Documents receiving this certification should be English-ready for publication; however, the author has the ability to accept or reject our suggestions and changes. To verify the final AJE edited version, please visit our verification page at aje.com/certificate. If you have any questions or concerns about this edited document, please contact AJE at support@aje.com.

AJE provides a range of editing, translation, and manuscript services for researchers and publishers around the world.
For more information about our company, services, and partner discounts, please visit aje.com.
